# Everyday Functioning in a Community-Based Volunteer Population: Differences Between Participant- and Study Partner-Report

**DOI:** 10.3389/fnagi.2021.761932

**Published:** 2022-01-05

**Authors:** Merike Verrijp, Mark A. Dubbelman, Leonie N. C. Visser, Roos J. Jutten, Elke W. Nijhuis, Marissa D. Zwan, Hein P. J. van Hout, Philip Scheltens, Wiesje M. van der Flier, Sietske A. M. Sikkes

**Affiliations:** ^1^Alzheimer Center Amsterdam, Department of Neurology, Amsterdam Neuroscience, Vrije Universiteit Amsterdam, Amsterdam UMC, Amsterdam, Netherlands; ^2^Division of Clinical Geriatrics, Center for Alzheimer Research, Department of Neurobiology, Care Sciences and Society, Karolinska Institutet, Stockholm, Sweden; ^3^Department of General Practice and Medicine for Older Persons, Amsterdam Public Health Research Institute, Amsterdam University Medical Center, Vrije Universiteit Amsterdam, Amsterdam, Netherlands; ^4^Department of Epidemiology and Biostatistics, Amsterdam UMC, Amsterdam, Netherlands; ^5^Faculty of Behavioural and Movement Sciences, Clinical Developmental Psychology, Clinical Neuropsychology, Vrije Universiteit Amsterdam, Amsterdam, Netherlands

**Keywords:** instrumental activities of daily living, aging, preclinical, awareness, Alzheimer’s disease, dementia, self report measures, study partner-reported outcomes

## Abstract

**Introduction:** Impaired awareness in dementia caused by Alzheimer’s disease and related disorders made study partner-report the preferred method of measuring interference in “instrumental activities of daily living” (IADL). However, with a shifting focus toward earlier disease stages and prevention, the question arises whether self-report might be equally or even more appropriate. The aim of this study was to investigate how participant- and study partner-report IADL perform in a community-based volunteer population without dementia and which factors relate to differences between participant- and study partner-report.

**Methods:** Participants (*N* = 3,288; 18–97 years, 70.4% females) and their study partners (*N* = 1,213; 18–88 years, 45.8% females) were recruited from the Dutch Brain Research Registry. IADL were measured using the Amsterdam IADL Questionnaire. The concordance between participant- and study partner-reported IADL difficulties was examined using intraclass correlation coefficient (ICC). Multinomial logistic regressions were used to investigate which demographic, cognitive, and psychosocial factors related to participant and study partner differences, by looking at the over- and underreport of IADL difficulties by the participant, relative to their study partner.

**Results:** Most A-IADL-Q scores represented no difficulties for both participants (87.9%) and study partners (89.4%). The concordance between participants and study partners was moderate (ICC = 0.55, 95% confidence interval [CI] = [0.51, 0.59]); 24.5% (*N* = 297) of participants overreported their IADL difficulties compared with study partners, and 17.8% (*N* = 216) underreported difficulties. The presence of depressive symptoms (odds ratio [OR] = 1.31, 95% CI = [1.12, 1.54]), as well as memory complaints (OR = 2.45, 95% CI = [1.80, 3.34]), increased the odds of participants overreporting their IADL difficulties. Higher IADL ratings decreased the odds of participant underreport (OR = 0.71, 95% CI = [0.67, 0.74]).

**Conclusion:** In this sample of community-based volunteers, most participants and study partners reported no major IADL difficulties. Differences between participant and study partner were, however, quite prevalent, with subjective factors indicative of increased report of IADL difficulties by the participant in particular. These findings suggest that self- and study partner-report measures may not be interchangeable, and that the level of awareness needs to be considered, even in cognitively healthy individuals.

## Introduction

As the research field of Alzheimer’s disease (AD) shifts its attention to earlier stages of the disease, clinically meaningful outcome measures that show early changes are becoming increasingly important (Edgar et al., 2019). One such outcome measure is the concept of “instrumental activities of daily living” (IADL), which refers to cognitively complex everyday activities (Lawton and Brody, 1969). Previous studies have shown that study partners report a decline in IADL in preclinical AD, even before cognitive problems can be detected by the standard cognitive testing (Sperling et al., 2011; Marshall et al., 2012, 2017; Zoller et al., 2014). Due to impairments in awareness in persons with dementia (Hanseeuw et al., 2020), (I)ADL functioning has traditionally been assessed using study partner-report questionnaires (Loewenstein et al., 2001; Howorth and Saper, 2003; Wadley et al., 2003; Desai et al., 2004; Farias et al., 2005; Graham et al., 2005; Sikkes et al., 2009; Hackett et al., 2020).

However, it has been suggested that study partner-report may be biased, by factors such as depression, anxiety, and caregiver burden (Zanetti et al., 1999; Arguelles et al., 2001; Ready et al., 2004). With a shift toward studying cognitively normal or “at-risk” individuals, one might assume that participants are able to reliably reflect on their own level of functioning, as they are thought to have accurate or potentially heightened awareness of their functional and cognitive abilities, as reflected in the concept of subjective cognitive decline (SCD) (Steward et al., 2019; Hanseeuw et al., 2020). In such populations, participant-report may therefore be a more appropriate and direct assessment method (DeBettignies et al., 1990; Zanetti et al., 1999; Arguelles et al., 2001).

When investigating participant- and study partner-report, a few findings stand out. First, several studies have found that there is no perfect concordance between participants and study partners, even in cognitively normal populations (Farias et al., 2005; Okonkwo et al., 2008; Marshall et al., 2020). Factors such as participant education, depression, and anxiety, as well as the nature of the relationship and the frequency and intensity of contact between participants and study partners, may affect how either party reports impairments, leading to discordance where one may report more or fewer impairments than the other. Second, studies investigating the interplay of these factors in cognitively normal populations are scarce. Furthermore, findings are difficult to compare between studies, due to differences in IADL measurements and in the definition and operationalization of concordance and discordance.

The Amsterdam IADL Questionnaire (A-IADL-Q) was developed as a study partner-rated questionnaire and has been extensively validated in memory clinic and community-based international aging populations (Sikkes et al., 2012, 2013a,b; Koster et al., 2015; Jutten et al., 2017; Facal et al., 2018; Villeneuve et al., 2019; Bruderer-Hofstetter et al., 2020; Dubbelman et al., 2020a). It is not yet known how the participant-report version of the A-IADL-Q performs and how it relates to study partner-report. The aim of this study was to investigate how the participant- and study partner-reported versions of the A-IADL-Q perform in a community-based population, without dementia, and what factors relate to differences between participant- and study partner-reported IADL functioning.

## Materials and Methods

### Participant Selection and Study Design

Participants were selected through the Dutch Brain Research Registry (Hersenonderzoek.nl), which is an online platform for people interested in cognition and brain-related research (Zwan et al., 2021). All eligible registrants were invited by email to participate in the study. The only inclusion criterion was participants being 18 years or older. Those who self-reported to have received a dementia-related diagnosis (i.e., dementia or mild cognitive impairment [MCI]) were excluded.

Data collection started in August 2018 and ended in December 2018. The study was approved by the medical ethical committee of the VU University Medical Center. The participants provided consent *via* Hersenonderzoek.nl. Since study partners were not recruited through Hersenonderzoek.nl, they provided consent prior to completing the online IADL questionnaire.

### Measures

#### Amsterdam Instrumental Activities of Daily Living Questionnaire

The main outcome measure was the A-IADL-Q. The A-IADL-Q was developed as a study partner-report instrument aimed at measuring problems in cognitively complex everyday functioning (Sikkes et al., 2012). For the current study, we adapted the study partner-report version to a participant-report version. Both versions consist of the same 30 items, covering a broad range of cognitive IADL. Each item assesses difficulty performing an activity due to cognitive problems, such as problems with memory, attention, or executive functioning. Item responses were rated on a five-point Likert scale, ranging from “no difficulty in performing this activity” (0) to “no longer able to perform this activity” (4). The total score is calculated using item response theory (IRT), assuming a single underlying construct (Reise and Waller, 2009), that is, IADL functioning, ranging from disability to ability. Total scores range from 20 to 70 and were reversed so that higher scores reflect better IADL functioning. A cutoff value for dementia was previously placed at 51.4 (Sikkes et al., 2013b), while scores above 60 were considered to indicate no IADL difficulties (Dubbelman et al., 2020b). The study partner-report version of the A-IADL-Q has undergone extensive validation, showing a good content and construct validity, high internal consistency, high test-retest reliability, good responsiveness to change and ablity to measure IADL across cultures and languages (Sikkes et al., 2013a,b; Koster et al., 2015; Jutten et al., 2017; Dubbelman et al., 2020a). The study partner version of the A-IADL-Q also includes questions about the type of relation to the participant and cohabitation. Study partners were classified as spouses, children, siblings, or “other.” Study partners in the “other” category included friends, coworkers, or other family members.

#### Other Measures

Cognitive functioning was assessed using the Cognitive Online Self-Test Amsterdam (COST-A), an online cognitive self-test developed and validated by Van Mierlo et al. (2017). The COST-A included 10 tasks, namely, orientation, digit-sequence learning, immediate word recall, two trail-making tasks (i.e., connecting numbered dots and alternately connecting lettered and numbered dots), delayed word recall, delayed word recognition, immediate recall of word pairs, recognition of word pairs, and semantic comprehension. Performance on each of the tasks was standardized and averaged into a *Z*-score to represent overall cognitive functioning, where higher scores indicate better cognition. Visser et al. (2021) provided a more detailed description of the COST-A.

In addition, a single yes/no question (“Do you have memory complaints?”) assessed subjective memory complaints. Depressive symptoms were assessed with the five-item short form of the Geriatric Depression Scale (GDS5) (Hoyl et al., 1999) with higher scores indicating more depressive symptoms. The education level was classified as low-medium (up to high school) and high education (college degree).

### Defining Awareness of Instrumental Activities of Daily Living Functioning

In line with other studies, we defined concordance based on the discrepancy between participant- and study partner-report (Hanseeuw et al., 2020). Based on a previously determined clinically meaningful difference over time of 2.4 points, we categorized concordance into three groups, (Dubbelman et al., 2020) namely, (1) concordance between dyads, (2) discordance between dyads with the participant “overreporting” difficulties (i.e., scoring ≥ 2.4 points lower than their study partner), and (3) discordance between dyads with the participant “underreporting” difficulties (i.e., scoring ≥ 2.4 points higher than their study partner).

### Statistical Analyses

Demographic differences between study partners and participants were tested using independent *t*-tests or chi-square tests. The frequency of IADL difficulties among cognitively normal participants and their study partners was determined. Then, in separate linear regression analyses, A-IADL-Q scores of both raters were associated with age, education, objective cognitive functioning, subjective cognitive functioning, and depressive symptoms.

The intraclass correlation coefficient (ICC) was computed to examine the absolute agreement between participant and study partner ratings. According to the criteria suggested by Koo et al., an ICC < 0.5 shows poor agreement, an ICC of 0.5–0.75 shows moderate, and an ICC > 0.75 shows good agreement (Koo and Li, 2016).

Using stepwise multinomial logistic regression models with backward selection, we investigated which factors related to concordance and discordance between dyads. The variables included the following parameters of participants: education level, sex, age, COST-A scores, memory complaints, GDS5 total score, study partner-reported IADL functioning, the type of relationship, cohabitation (yes/no), and the absolute age difference between dyads. For this analysis, COST-A scores were dichotomized into normal (more than -1.5 SD) and low (less than or equal to −1.5 SD) cognitive functioning. All analyses were performed using R version 4.0.3 software (R Core Team, 2020).

## Results

Of the 11,060 eligible registrants, 4,817 individuals (44%) were interested in participation and received study instructions. After receiving instructions, 3,288 (68%) individuals completed the participant-reported A-IADL-Q. On average, participants were 61.0 ± 12.1 years old and the majority of them were women (i.e., 2,315; 70.4%). Approximately, half the participants experienced memory complaints. Table 1 displays all participant and study partner characteristics. Participant and study partner characteristics stratified by age groups are shown in Supplementary Material.

**TABLE 1:** Participant and study partner characteristics.

	Participants (*N* = 3,288)	Dyads (*N* = 1,213)	
		Participants	Study partners
Age, mean (SD)	61.0 (12.1)	62.5 (11.1)	58.8 (14.2)
Range	18–97	18–93	18–88
Female, n (%)	2,315 (70.4)	828 (68.3)	556 (45.8)
High level of education, n (%)	2,323 (70.7)	854 (70.4)	—
A-IADL-Q score, mean (SD)	65.9 (4.8)	65.9 (4.7)	66.1 (4.6)
Range	40.9–70.0	40.7–70.0	42.7–70.0
Memory complaints present,^1^ n (%)	1,429 (47.5)	586 (49.9)	—
COST-A,^2^ abnormal performance (≤ -1.5SD), n (%)	225 (7.6)	86 (7.5)	—
GDS5,^1^ median (IQR)	0 (0–1)	0 (0–1)	—
**Type of relationship, n (%)** Spouse Child Sibling Other		956 (78.8) 155 (12.8) 32 (2.6) 70 (5.8)	
**Duration relationship, n (%)** < 5 years 5–10 years >10 years		33 (2.7) 58 (4.8) 1,119 (92.5)	
Living together, n (%)		960 (79.3)	

*“—” denotes that the data were not available. ^1^Data were available for 3,011 participants, of whom 1,175 were part of a dyad. ^2^Data were available for 2,945 participants, of whom 1,149 were part of a dyad. A-IADL-Q, Amsterdam Instrumental Activities of Daily Living Questionnaire; COST-A, Cognitive Self-Test Amsterdam; GDS5, 5-item Geriatric Depression Scale; IQR, interquartile range; SD, standard deviation.*

For 1,213 participants (36.9% of complete sample), the A-IADL-Q was also completed by a study partner (participant and study partner pairs will be referred to as “dyads”). Participants who were part of a dyad were 62.5 ± 11.1 years old, and the majority of them were women (i.e., 828; 68.3%). They were older (*p* < 0.001) and more often men (*p* = 0.046) than participants who were not part of a dyad. Within dyads, the participants were older (*p* < 0.001) and more likely to be women (*p* < 0.001) than study partners.

### Instrumental Activities of Daily Living Difficulties in a Cognitively Normal Population

**FIGURE 1:**
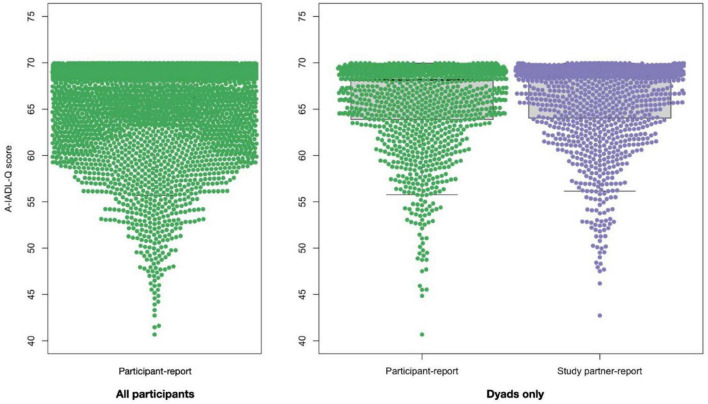
A-IADL-Q total score distribution among all participants (left panel, *N* = 3,288) and among dyads (right panel, *N* = 1,213, participants are denoted in green and study partners are denoted in purple).

Figure 1 shows the distribution of participant- and study partner-reported A-IADL-Q scores. Among dyads, the participant-reported A-IADL-Q scores (65.9 ± 4.8) did not differ from the study partner-reported A-IADL-Q scores (66.1 ± 4.6; *p* = 0.186). Virtually all participants (3,232/3,288; 98.3%) and study partners (1,195/1,213; 98.5%) reported A-IADL-Q scores above a previously established cutoff for dementia (total score of 51.4). Moreover, the vast majority of both participant-reported (87.9%) and study partner-reported (89.4%) total scores were higher than 60, indicating no difficulties.

**FIGURE 2:**
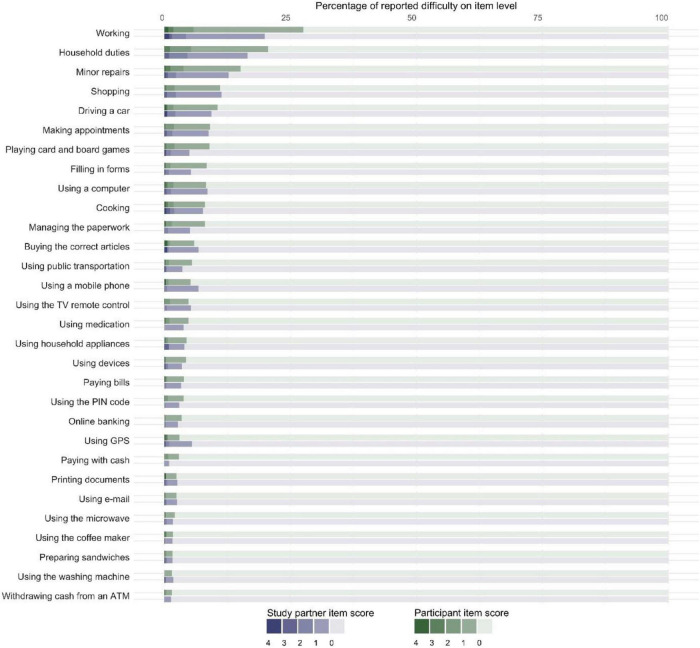
Stacked bar chart showing the percentage of participants (denoted in shades of green) and study partners (denoted in shades of purple) who reported difficulties (*N* = 1,213). The dark shades represent difficulty with the activity: “no longer able to perform this activity” (4), “much more difficulty” (3), “more difficulty” (2), and “slightly more difficulty” (1). The lightest shade represents “no difficulty in performing this activity” (0). Displaying data from dyads only.

Then, we examined IADL difficulties at an item level. Half of all participants (i.e., 1,750/3,288, 53.2%) and study partners (i.e., 722/1,213, 59.5%) reported no difficulties in any activity. Those who reported difficulties mostly did so in only one activity (i.e., 35.2% of participants and 35.8% of study partners). Figure 2 shows the percentage of participants and study partners who reported difficulties for each IADL activity. Most frequently reported IADL difficulties for both participants and study partners were working (i.e., 26.9 and 19.9%, respectively), household duties (i.e., 22.2 and 16.5%, respectively), and making minor repairs at home (i.e., 16.4 and 12.7%, respectively).

Table 2 shows the associations between age, education level, cognitive complaints, COST-A, GDS, and participant- and study partner-reported IADL performance. Higher age was associated with lower A-IADL-Q scores, and higher education was associated with better A-IADL-Q scores, but associations were weak. For example, with every 10 years increase in age, A-IADL-Q participant- and study partner-reported scores decreased with 1.2 and 1.8 points, respectively. Both participant- and study partner-reported A-IADL-Q scores were more highly associated with COST-A scores, memory complaints, and GDS. Higher COST-A scores, indicating better cognitive functioning, were associated with better IADL functioning, whereas a higher GDS, indicating more depressive symptoms, and presence of memory complaints were associated with worse IADL functioning. Associations with age, education, and COST-A scores were comparable for participant- and study partner-report, whereas associations with GDS and memory complaints were more strongly associated with participant-reported IADL scores.

**TABLE 2:** Linear regressions to investigate associations with participant- and study partner-reported IADL performance.

Measure	Participant-report	Study partner-report
Age	−0.12 [−0.16, −0.09]	−0.18 [−0.26, −0.14]
High education	0.09 [0.06, 0.13]	0.07 [0.02, 0.13]
Memory complaints present	−0.33 [−0.36, −0.29]	−0.24 [−0.30, −0.19]
COST-A	0.23 [0.19, 0.26]	0.25 [0.20, 0.31]
GDS5	−0.33 [−0.36, −0.29]	−0.21 [−0.30, −0.17]

*Associations are shown as standardized beta [95% confidence interval]. Some measures were not available for the entire sample. Memory complaints were available for N = 3,011 participants and N = 1,175 participants who were part of a dyad. COST-A scores were available for N = 2,945 participants and N = 1,149 participants who were part of a dyad. GDS5 scores were available for N = 3,017 participants and N = 1,177 participants who were part of a dyad.*

### Concordance and Discordance Between Dyads

There was a moderate agreement between participant- and study partner-reported IADL functioning (ICC = 0.55, 95% CI = [0.51, 0.59], *p* < 0.001; see Supplementary Table 1). Of all 1,213 dyads, 700 (57.7%) were in concordance. Two hundred sixteen participants (17.8%) underreported difficulties, compared with their study partners, and 297 participants (24.5%) overreported IADL difficulties, compared to their study partners. Compared with concordant dyads, participants with memory complaints (odds ratio [OR] = 2.44, 95% CI = [1.80, 3.32], *p* < 0.001) and with a higher GDS (OR = 1.31, 95% CI = [1.12, 1.53], *p* = 0.001) were more likely to overreport IADL difficulties (see Table 3). Participant underreport was less likely when there were fewer IADL difficulties (OR = 0.71, 95% CI = [0.67, 0.74], *p* < 0.001). Thus, concordance was more likely when the participant did not experience memory complaints, when they had lower GDS scores, and when IADL performance was higher. Education, age, gender, and COST-A scores of participants were not related to concordance between dyads.

**TABLE 3:** Multivariable multinomial logistic regression models comparing study partners reporting more IADL difficulties than participant (*N* = 216) and participant reporting more IADL difficulties than study partner (*N* = 297), compared with agreement between the participant and study partner (*N* = 700).

Predictor	Study partner > Participant (*N* = 216)		Participant > study partner (*N* = 297)	
	OR [95% CI]	*P*	OR [95% CI]	*P*
COST-A ≤ -1.5 SD	0.47 [0.21, 1.07]	0.070	1.36 [0.78, 2.39]	0.283
A-IADL-Q (study partner-report)	0.71 [0.67, 0.74]	<0.001	1.04 [0.99, 1.09]	0.148
Memory complaints present	0.76 [0.50, 1.15]	0.194	2.44 [1.80, 3.32]	<0.001
High education	0.92 [0.60, 1.40]	0.689	1.30 [0.93, 1.80]	0.121
Absolute age difference between dyads in years	1.00 [0.97, 1.04]	0.924	1.01 [0.98, 1.04]	0.924
Age in years (participant)	1.01 [0.99, 1.03]	0.467	1.01 [0.99, 1.02]	0.272
Female sex (participant)	0.74 [0.53, 1.02]	0.159	1.08 [0.78,1.49]	0.661
GDS5*	0.58 [0.50, 0.68]	<0.001	1.31 [1.12, 1.53]	<0.001
**Type of relationship, study partner is a:^†^** Child Sibling Other	2.19 [0.63, 7.60] 0.75 [0.13, 4.35] 0.81 [0.22, 2.98]	0.216 0.744 0.755	0.83 [0.30, 2.27] 0.57 [0.18, 1.85] 0.63 [0.24, 1.68]	0.716 0.350 0.355
Dyads live together	1.58 [0.70, 3.57]	0.277	1.04 [0.57, 1.90]	0.898

OR, odds ratio; 95% CI, 95% confidence interval. Concordance was used as a reference group (N = 700). *More depressive symptoms; ^†^Using spouse as a reference category.

## Discussion

In this study, we showed that the majority of IADL scores fell within the range of normal IADL functioning in this community-based population, but that discordance among dyads was quite prevalent. A small proportion reported subtle IADL difficulties, which was associated with older age, lower education, worse cognitive performance, presence of self-reported memory complaints, and more depressive symptoms of participants, for both participant- and study partner-report. A moderate agreement between participant- and study partner-reported IADL was found with discordance between dyads being more likely when the participant reported memory complaints, and had depressive symptoms and lower IADL performance.

While the large majority of participant- and study partner-reported IADL functioning fell within the range of normal IADL functioning, approximately a tenth of both participants and study partners scored below the previously established cutoff for normal IADL functioning (Dubbelman et al., 2020b). This prevalence of impaired IADL is comparable to other population-based studies (Ostbye et al., 1997; Pudaric et al., 2003; Crimmins et al., 2011; Scheel-Hincke et al., 2020). For example, Scheel-Hincke et al. (2020) reported a prevalence of impaired IADL of 12 to 20% in Western Europe, with impaired IADL defined as presence of any difficulties. Another population-based study by Pudaric et al. (2003) reported a prevalence of impaired IADL (inability to carry out shopping, cooking, or housework) of 6 to 11%. Despite this comparable prevalence of abnormal IADL functioning, it is important to note that approximately half of our population reported more subtle difficulties. If we applied the definition of Scheel-Hincke et al. (2020), the prevalence of impaired IADL in our study would be approximately 50%, which is substantially higher than the prevalence that they reported. There are two potential explanations for this difference: first, we included more activities, and second, and more importantly, we included more cognitively complex activities than other studies. This is illustrated by the fact that most problems were reported in working, household duties, and making repairs, which are especially cognitively complex (Jutten et al., 2017). These activities were not included in other IADL scales. For example, a population-based study that assessed five IADL items (Chan et al., 2012) reported most problems for shopping. In our population, problems with shopping were fourth most prevalent. We found a higher proportion of difficulties for more complex activities, supporting the notion that including more complex activities enabled detection of more fine-grained difficulties in IADL functioning.

With regard to potential sources of bias in the report of IADL functioning, we found low associations between both study partner- and participant-reported IADL functioning and age and education. This finding is supported by previous validation studies for the study partner version of the A-IADL-Q (Sikkes et al., 2013a; Jutten et al., 2017; Dubbelman et al., 2020a). Participant- and study partner-report were similarly associated with objective cognitive performance, but participant-reported IADL functioning was more strongly related to depressive symptoms, as well as subjective cognitive performance (i.e., presence of self-reported memory complaints). Consistent with recent literature suggesting that study partners are better able to assess the functioning of participants than the participant themselves (Howland et al., 2017), our findings might imply that study partner-report is less biased than participant-report by participant-related subjective factors.

Our findings demonstrated only a moderate concordance between dyads. While the distributions of study partner- and participant-reported IADL scores were largely similar, we found a moderate ICC and a high proportion of discordance (either over- or underreport). Other studies have also shown discordance in cognitively normal participants and, specifically, participant overreport (Ostbye et al., 1997; Farias et al., 2005; Okonkwo et al., 2008; Pol et al., 2011). For example, a study by Okonkwo et al. (2008) showed slight discordance between participant- and study partner-report of specific finance-related IADL. The proportion of discordance that we found in our study is substantially higher, which is probably due to differences in IADL measures, definitions of concordance, and population differences. As opposed to Okonkwo et al. (2008), who calculated concordance based on an individual item, we determined concordance based on a more global measure of IADL with a wider range of activities. We calculated concordance based on a clinically meaningful difference in total scores. Another potential explanation may be that, even though we used a population-based sample, we did not screen for cognitive impairment. As such, it is possible that there were participants who had subtle cognitive impairment but did not meet criteria for MCI or dementia. Thus, while the proportion of discordance is difficult to compare with other studies, the fact that other studies also reported discordance suggests that participant- and study partner-report might not be interchangeable.

The potential limited interchangeability is further supported by our results, which indicate that concordance is influenced by self-reported memory complaints and depressive symptoms. Participants with memory complaints reported more difficulties, compared with their study partners. Participant overreport of memory complaints has previously been described as a heightened awareness (Hanseeuw et al., 2020), which is thought to characterize early stages of AD and related disorders (Jessen et al., 2014; Slot et al., 2019; Hanseeuw et al., 2020). Following this theory, a subgroup of our study sample may have a heightened functional awareness. This idea is further supported by our finding that a large proportion of our sample had memory complaints, which may indicate a heightened memory awareness. While no other studies have investigated the effect of subjective cognitive functioning on the concordance of functional impairment, several studies (Weinberger et al., 1992; Ostbye et al., 1997; Albert et al., 1999; Tabert et al., 2002; Farias et al., 2005; Okonkwo et al., 2008; Pol et al., 2011) related objective cognitive functioning to concordance. These studies show that patients with poorer global cognition are more likely to underreport IADL difficulties. We did not find a significant association between concordance and objective cognition within our healthy volunteer population. This could be due to the fact that our population is presumably cognitively healthy, and lowered awareness may not occur until cognitive problems start to develop (Starkstein et al., 2006; Hanseeuw et al., 2020). Although not significant, in this population, lower cognitive performance seems to be related to reduced odds for participant underreport. This might suggest that the subtle cognitive problems of these individuals do not interfere with their disease insight, but rather, that they increase their awareness. Furthermore, participants with depressive symptoms were more likely to overreport, and less likely to underreport, IADL difficulties. This was also reported in studies in MCI and dementia that showed a greater chance of discordance when participants had depressive symptoms (Magaziner et al., 1996; Okonkwo et al., 2008). This is in line with the idea that negative self-perception in patients with depressive symptoms causes exaggeration of deficits (Lahr et al., 2007), as has also been shown by Okonkwo et al. (2008), who reported that underestimation of financial abilities was related to higher depressive symptoms. Thus, memory complaints and depressive symptoms both influence the report of IADL difficulties of participants and need to be taken into consideration when using participant-reported IADL measures.

The findings discussed earlier may have important implications for study design decisions and should be considered carefully when considering the use of a participant-reported IADL instrument. Although a concordance of 60% might seem low, the majority of both participant- and study partner-reported difficulties fell within the category of “no difficulties.” This crude overlap indicates that participant-report IADL can be useful in cognitively normal populations in cross-sectional studies. However, when a deterioration of cognitive functioning and subsequently everyday functioning is to be expected, study partner-report might provide a more reliable indication of change in IADL functioning. The combination of participant- and study partner-report can be used to establish awareness, which is informative since it has been shown to predict future disease progression (Nosheny et al., 2019, 2020) and greater discordance seems to be related to a greater risk of Alzheimer pathology (Tabert et al., 2002; Hanseeuw et al., 2020). The combination of participant- and study partner-report might also be valuable as they seem to reflect different perspectives. This is reflected in the current study as participant-report seems to be more influenced by subjective factors than the study partner-report. The different perspectives were also implied in an article by Amariglio et al. (2021) who showed that distinct IADL items were related to amyloid pathology for participants and study partners. Thus, participant self-report can be used in cognitively normal populations but should ideally be supplemented by study partner-report, not only when considering the cognitive decline of participants in longitudinal studies but also to gain multiple perspectives and insight into the awareness of participants.

Some limitations should be considered when interpreting our findings. For the lack of an objective IADL measure, we cannot ascertain whether participants indeed overreport their difficulties or whether participants actually have IADL difficulties that the study partner does not yet notice. In contrast, a heightened participant awareness may also reflect lowered study partner awareness. This caveat notwithstanding, the absence of an association between participant overreport and objective cognitive functioning could indicate that participant overreport is more strongly influenced by subjective than objective factors. It should also be noted that objective cognition and IADL performance cannot be completely separated, as IADL performance is dependent on cognition. This may introduce some level of circularity into the analyses. However, the association between our objective cognitive measure and the A-IADL-Q scores was only moderate. Furthermore, as the study partner-report is generally considered a gold standard in dementia research and clinical practice (Sikkes and De Rotrou, 2014), we used it as such in the current study. Another limitation is the selective nature of the volunteer registry, which consists mostly of highly educated and highly motivated individuals. This may limit generalizability to the general population. We did not include factors such as caregiver burden, personality traits, or more detailed information on the amount of contact between the participant and the study partner. Future studies should consider assessing these factors to obtain more detailed insight into the accuracy of assessments and possible biases. Furthermore, follow-up studies are needed to determine the pivot point until which the participant is still able to reliably evaluate their own level of daily functioning.

An important strength of this study is the large sample of cognitively healthy volunteers, representing a large range of ages, from early adulthood to late life. We included detailed information about the level of IADL difficulties from both self- and study partner-report in a cognitively healthy population, providing valuable new insights into the occurrence of more subtle IADL difficulties. While the clinically meaningful cutoff was determined for decline and not for differences between respondents, a strength of this clinically meaningful cutoff to distinguish concordance from discordance is that we believed that discordance actually represented an important, non-negligible difference in IADL report.

## Conclusion

Our findings show a moderate concordance between participants and study partners in reporting IADL difficulties, with subjective factors influencing the level of concordance. These findings suggest caution in using self- and study partner-report measures interchangeably, even in cognitively healthy community-based samples. Our results suggest that participant report might be more related to subjective factors and that study partner-report is less associated with these factors, possibly reflecting differing perspectives.

## Data Availability Statement

The raw data supporting the conclusions of this article will be made available by the authors, without undue reservation.

## Ethics Statement

The studies involving human participants were reviewed and approved by the VU University Medical Center. The participants provided their written informed consent to participate in this study.

## Author Contributions

SS, WF, HH, MV, and MZ: conception or design of the work. MZ, EN, LV, and MV: data collection. MV, MD, SS, WF, and HH: data analysis and interpretation. SS, MD, MV, and LV: drafting the manuscript. All authors provided critical revision of the article and final approval of the version to be published.

## Conflict of Interest

The authors declare that this study received funding from Stichting Stoffels-Hornstra. The funder was not involved in the study design, collection, analysis, interpretation of data, the writing of this article or the decision to submit it for publication.

## Publisher’s Note

All claims expressed in this article are solely those of the authors and do not necessarily represent those of their affiliated organizations, or those of the publisher, the editors and the reviewers. Any product that may be evaluated in this article, or claim that may be made by its manufacturer, is not guaranteed or endorsed by the publisher.
