# Supplementary material for: Everyday Functioning in a Community-Based Volunteer Population: Differences Between Participant- and Study Partner-Report
Source: Front Aging Neurosci. 2022 Jan 5;13:761932. doi: 10.3389/fnagi.2021.761932 (PMC8767803; doi:10.3389/fnagi.2021.761932)
Supplement: Supplementary file 1 [file Data_Sheet_1.docx]

# Supplementary Material

Supplementary Table 1 displays the characteristics of participants in the entire sample, as well as characteristics of participants from dyads and study partners, stratified by age group. The age groups are based on the participant’s age, with the following groups: participants younger than 40 years of age, participants aged 40 through 49 years, participants aged 50 through 59 years, participants aged 60 through 69 years and participants aged 70 years and older.

Supplementary Table 1 Participant and study partner characteristics, by age group

|  | **Participants** | **Dyads** | |  |
| --- | --- | --- | --- | --- |
|  |  | **Participants** | **Study partners** |  |
| **Age groups, n (%)^a^**  Under 40  40-49  50-59  60-69  Over 70 | **3,288 (100.0)**  189 (5.7)  238 (7.2)  883 (26.9)  1,224 (37.2)  754 (22.9) | **1,213 (100.0)**  46 (3.8)  63 (5.2)  295 (24.3)  504 (41.5)  305 (25.1) | **58.8 (14.2)**  38.3 (13.0)  46.6 (12.3)  51.4 (13.3)  61.5 (10.8)  67.0 (12.6) |  |
| **Female, n (%)**  Under 40  40-49  50-59  60-69  Over 70 | **2,315 (70.4)**  149 (78.8)  179 (75.2)  696 (78.8)  871 (71.2)  420 (55.7) | **828 (68.3)**  37 (80.4)  49 (77.8)  217 (73.6)  362 (71.8)  163 (53.4) | **556 (45.8)**  18 (39.1)  24 (38.1)  118 (40.0)  204 (40.5)  192 (63.0) |  |
| **High level of education, n (%)**  Under 40  40-49  50-59  60-69  Over 70 | **2,323 (70.7)**  169 (89.4)  180 (75.6)  647 (73.2)  814 (66.5)  513 (68.0) | **854 (70.4)**  43 (93.5)  46 (73.0)  209 (70.8)  343 (68.1)  213 (69.8) | — |  |
| **A-IADL-Q score, mean (SD)**  Under 40  40-49  50-59  60-69  Over 70 | **65.9 (4.8)**  67.1 (4.2)  66.1 (4.8)  66.3 (4.6)  65.8 (4.8)  65.0 (4.9) | **65.9 (4.7)**  67.3 (5.1)  66.0 (4.5)  66.2 (4.9)  66.0 (4.5)  65.2 (4.7) | **66.1 (4.6)**  68.1 (3.3)  67.1 (3.6)  66.6 (4.7)  66.2 (4.4)  65.1 (4.9) |  |
| **Memory complaints, n (%)**  Under 40  40-49  50-59  60-69  Over 70 | **1,429/3,011 (47.5)**  36/175 (20.6)  98/225 (43.6)  355/818 (43.4)  574/1,137 (50.5)  366/656 (55.8) | **586/1,175 (49.9)**  4/46 (8.7)  27/61 (44.3)  143/285 (50.2)  249/493 (50.5)  163/290 (56.2) | — |  |
| **Abnormal performance (≤ -1.5SD) on COST-A, n (%)**  Under 40  40-49  50-59  60-69  Over 70 | **218/2,945 (7.4)**  0/173 (0.0)  7/223 (3.1)  26/805 (3.2)  77/1,103 (7.0)  108/631 (17.1) | **83/1,149 (7.2)**  0/45 (0.0)  1/60 (1.7)  14/283 (4.9)  23/484 (4.8)  45/277 (16.2) | — |  |
| **GDS5, median (IQR)**  Under 40  40-49  50-59  60-69  Over 70 | **0 (0–1)**  0 (0–1)  0 (0–1)  0 (0–1)  0 (0–1)  0 (0–1) | **0 (0–1)**  0 (0–1)  0 (0–1)  0 (0–1)  0 (0–1)  0 (0–1) | — |  |
| **Dyads are spouses, n (%)**  Under 40  40-49  50-59  60-69  Over 70 | — | **956 (78.8)**  30 (65.2)  47 (74.6)  233 (79.0)  424 (84.1)  222 (72.8) | | |
| **Duration relationship >10 years, n (%)**  Under 40  40-49  50-59  60-69  Over 70 | — | **1,119 (92.5)**  28 (60.9)  56 (88.9)  265 (89.8)  479 (95.0)  291 (95.4) | | |
| **Living together, n (%)**  Under 40  40-49  50-59  60-69  Over 70 | — | **960 (79.3)**  29 (63.0)  51 (81.0)  250 (84.7)  418 (82.9)  212 (69.5) | | |

^a^ For study partners, the table displays the mean age (SD).

Abbreviations: A-IADL-Q, Amsterdam Instrumental Activities of Daily Living Questionnaire; COST-A, Cognitive Self-Test Amsterdam; GDS5, 5-item Geriatric Depression Scale; IQR, interquartile range; SD, standard deviation.

## Relationship between participant and study partner-reported IADL scores


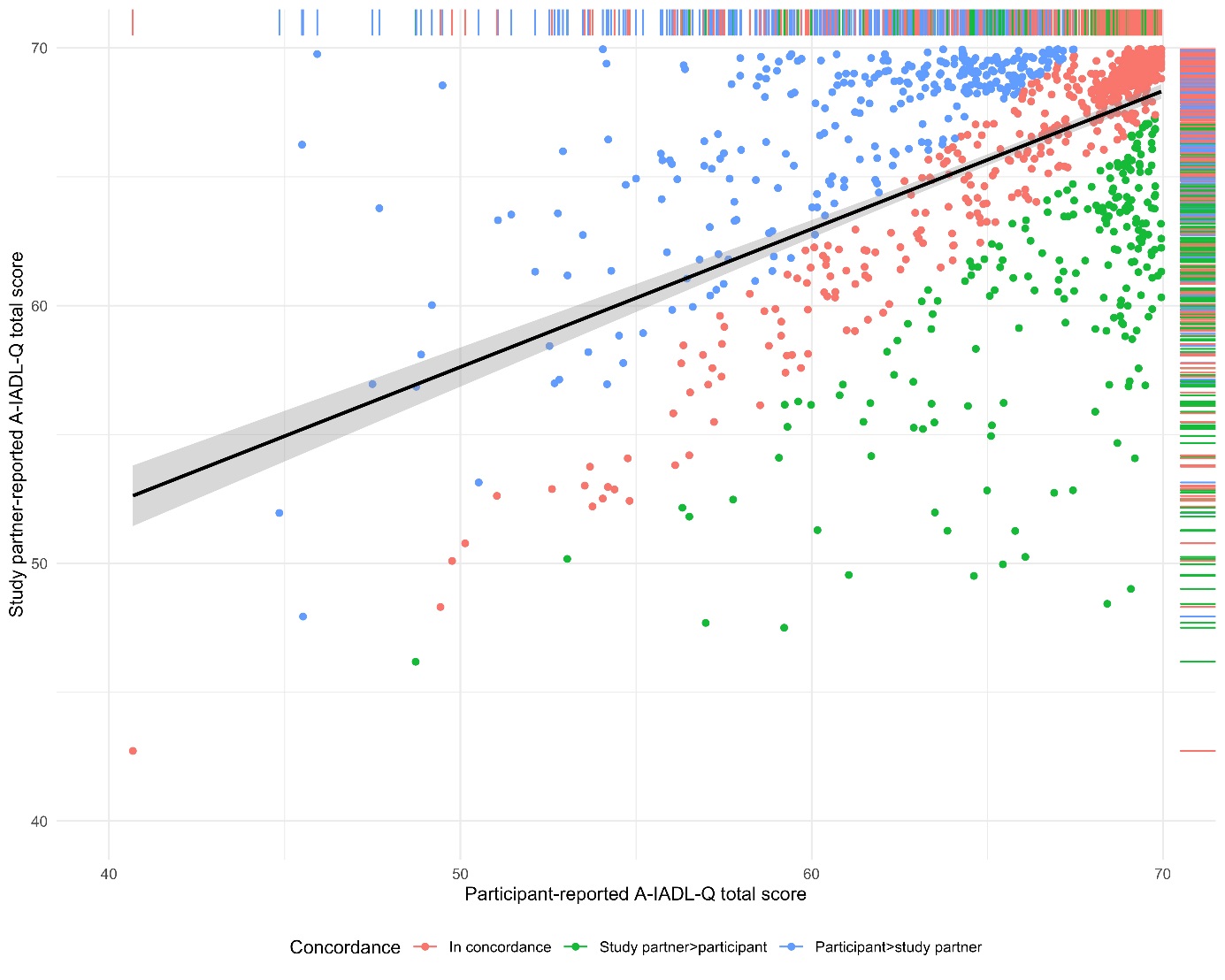


Figure 1. Scatterplot showing the relationship (black line) between participant-reported (horizontal axis) and study partner-reported IADL functioning (vertical axis).

Each dot represents an individual; dots are colored based on a difference in IADL-Q scores of 2.4 points or more: dyads in concordance are red, dyads where the study partner reported better A-IADL-Q scores than the participant are green, dyads where the participant reported better A-IADL-Q scores than the study partner are blue.
